# Supplementary material for: Quality of life and associated factors among Norwegian public health nurse students during the first months of the COVID-19 pandemic: A cross-sectional study
Source: PLoS One. 2023 Mar 16;18(3):e0282653. doi: 10.1371/journal.pone.0282653 (PMC10019636; doi:10.1371/journal.pone.0282653)
Supplement: Attachment — Submitted filename: Respons to the Reviewers.docx [file pone.0282653.s002.docx]

Reviewer #1: (No Response)

Reviewer #2: Most comments have been clarified and supplemented, however, in my opinion there are still several problems need to be improved.

**Comment 1:** The revised manuscript should be marked with different colors for reviewer.

**Response 1:** Thank you for giving us the opportunity to review this manuscript. Now, all changes made in both the previous revision and the present revision are highlighted in red.

**Comment 2:** In abstract section, the result of “The strongest predictive factors for QOL were found to be perceived loneliness and self-efficacy” should add “among these factors”.

**Response 2:** This is now included in the abstract page 2 line 42.

**Comment 3:** Observational research also needs to describe the criteria for inclusion and deletion. Observational study should comply with the STROBE (Strengthening the Reporting of Observational Studies in Epidemiology) statement

**Response 3:** We acknowledge the reviewer’s comments and have now clarified the criteria for inclusion in the study on page 5 and 6 line 129-136.

All students, both full-time and part-time, enrolled in PHN educational programme in eight Universities and Colleges in Norway during the COVID-19 pandemic met the inclusion criteria for this study. The participating universities were Oslo Metropolitan University, VID Specialized University, University of South-Eastern Norway (USN), the Norwegian University of Science and Technology (NTNU), University of Stavanger (UIS), The Arctic University of Norway (UIT), Inland Norway University of Applied Sciences and The Western Norway University of applied Sciences.

**Comment 4**: Additional protocol and reference on the measure of mental and physical health are needed.

**Response 4:**

We acknowledge the reviewer’s comments and references on the measures about mental and physical health are now included in the method section page 7 and 8.

The questions about mental and physical health are from the National Norwegian Recommendations for measuring subjective quality of life in Norway. The recommendations are based on a review of research and international recommendations, input from national and international actors in the field, as well as professional assessments of quality of life. (Nes, Hansen and Barstad, 2018). The Norwegian recommendations are based on the Dolan and Metcalf’ Recommendations on measures for use by national governments, which recommend that both physical and mental health be included as questions in measures of cognitive subjective quality of life. (Dolan and Metcalf, 2012). Both questions about mental and physical health have been tested and validated in a Norwegian setting (Berg, Berglund and Lund, (2018).

**Comment** 5: Measurement results or questionnaire score of QOL and civil status, self-efficacy, loneliness, and physical and mental health should be reported and displayed. I suggest adding another table to present these results and change Table 2 to Table 3.

**Response 5:** We agree with the reviewer’s comments. We have now added a new table with descriptive statistics on the questionnaire score of QOL, civil status, self-efficacy, loneliness, and physical and mental health. As the responses on physical and mental quality of life are continuous variables, the data are presented as median and ranges. The results are now included in table 2 on page 10.
